# Patients receiving hemodialysis do not lose SARS-CoV-2 antibodies more rapidly than non-renal controls: a prospective cohort study

**DOI:** 10.1080/0886022X.2022.2042310

**Published:** 2022-02-28

**Authors:** Ekaterina Parshina, Alexey Zulkarnaev, Alexey Tolkach, Andrey Ivanov, Pavel Kislyy, Abduzhappar Gaipov

**Affiliations:** a Department of Nephrology and Dialysis, Saint-Petersburg State University Hospital, Saint-Petersburg, Russia; b Surgical Department of Transplantology and Dialysis, Moscow Regional Research and Clinical Institute ("MONIKI"), Moscow, Russia; c Human Genetics Department, Saint Petersburg State University Hospital, Saint-Petersburg, Russian Federation; d Department of Medicine, Nazarbayev University School of Medicine, Nur-Sultan, Kazakhstan

**Keywords:** COVID-19, SARS-CoV-2, hemodialysis, antibody, cellular immunity

## Abstract

**Background:**

Patients with end-stage kidney disease receiving maintenance hemodialysis (HD) are at increased risk for mortality after infection with severe acute respiratory syndrome coronavirus 2 (SARS-CoV-2) compared with the general population. However, it is currently unknown whether the long-term SARS-CoV-2 humoral and cellular immune responses in patients receiving HD are comparable to individuals with normal kidney function.

**Method:**

The prospective cohort study included 24 patients treated with maintenance HD and 27 non-renal controls with confirmed history of coronavirus disease (COVID-19). In all participants the levels of specific IgG were quantified at three timepoints: 10, 18, and 26 weeks from disease onset. In a subgroup of patients, specific T-cell responses were evaluated.

**Results:**

The seropositivity rate declined in controls over time and was 85% and 70.4% at weeks 18 and 26, respectively. All HD patients remained seropositive over the study period. Seropositivity rate at week 26 was greater among patients receiving HD: RR = 1.4 [95%CI: 1.17–1.94] (reciprocal of RR = 0.7 [95% CI: 0.52–0.86]), *p* = 0.0064. In both groups, IgG levels decreased from week 10 to week 26, but antibodies vanished more rapidly in controls than in HD group (ANOVA *p* = 0.0012). The magnitude of T-cell response was significantly lower in controls than in HD patients at weeks 10 (*p* = 0.019) and 26 (*p* = 0.0098) after COVID-19 diagnosis, but not at week 18.

**Conclusion:**

Compared with non-renal adults, patients receiving HD maintain significant long-term humoral and cellular immune responses following natural COVID-19.

## Introduction

Patients with end-stage kidney disease (ESKD) receiving hemodialysis (HD) are vulnerable to coronavirus disease (COVID-19) because of multiple risk factors [1]. The multicenter ERACODA (European Renal Association COVID-19 Database) study found that the 28-day COVID-19-related mortality was 25% in all dialysis patients and 33.5% in those who required hospitalization, which is markedly higher than that in general population [2]. Better understanding the natural immunity to SARS-CoV-2 infection would be helpful in protecting these patients against re-infection through either implementation of isolation measures or development of vaccination policies.

Several studies have explored the duration of the humoral immune response after natural COVID-19 in patients receiving HD [3–5], although there remains a lack of knowledge about differences in severe acute respiratory syndrome coronavirus 2 (SARS-CoV-2) immunoglobulin G (IgG) dynamics in patients receiving dialysis compared with individuals with no underlying renal diseases. In addition, the evolution of the cellular response after COVID-19 in patients receiving HD over time has not been studied previously.

Considering this knowledge gap, we performed a prospective cohort study aimed to compare long-term SARS-CoV-2 humoral and cellular immune responses in patients with ESKD treated with maintenance HD (*n* = 24) and non-renal controls (*n* = 27) who had not received vaccination before and during the study period. We hypothesized that specific IgG antibodies against SARS-CoV-2 decline more rapidly in patients receiving HD than in controls. In addition, we assumed that the intensity of the T-cell response would be lower among dialysis-dependent subjects.

## Materials and methods

### Study population

This prospective cohort study was conducted from January to July 2021 at the Saint Petersburg State University Hospital. Fifty-one convalescent participants were enrolled in the study, 24 of whom were dialysis-dependent and 27 were non-renal volunteers. All patients received hemodialysis (HD) more than 6 months in a single unit of the Saint Petersburg State University Hospital, and controls were healthcare workers at the same hospital. There were no underlying renal diseases among healthcare workers based on data from routine annual examinations. The inclusion criteria were age of 18 years and older, a confirmed history of coronavirus disease (COVID-19) within 10 weeks prior to enrollment, and informed consent to participate in the study. The exclusion criteria were SARS-CoV-2 vaccination or re-infection. One patient receiving HD did not develop specific antibodies and was therefore excluded from the subsequent analyses. Three patients receiving HD were lost to follow-up after the first or second visit due to death or kidney transplantation, but their data were used in the analysis.

The diagnosis of COVID-19 was confirmed based on a positive real-time polymerase chain reaction (PCR) test using nasopharyngeal swabs and/or compatible findings on computed tomography scans of the lungs based on common CT guidelines [6]. Disease onset was set up as date of detecting first symptoms or date of first positive polymerase chain reaction result in cases of asymptomatic disease. The time for viral clearance was calculated as the interval between the disease onset and recovery based on the first negative nasopharyngeal swab.

Data were collected from the participants (age, sex, comorbidities, concomitant immunosuppressive therapy, COVID-related medical history), either from medical records or self-reports. Comorbidities were assessed using a modified cumulative illness rating scale (Cumulative Illness Rating Scale-Geriatric). All patients receiving HD (even those who were completely asymptomatic) were hospitalized regardless of the severity of the disease in accordance with the local isolation protocol, while nobody in the control group required hospitalization. There were no critical conditions or the need for mechanical ventilation among the hospitalized patients. Therefore, all the cases of COVID-19 were interpreted as mild or moderate.

### Study procedures

In all participants, the levels of anti-SARS-CoV-2-specific IgG were quantified at three time points: 10 weeks, 18 weeks, and 26 weeks from disease onset (i.e., date of first symptoms or from the date of positive PCR result in cases of asymptomatic disease). IgG levels were determined in venous blood using a semi-quantitative SARS-CoV-2 S1 IgG enzyme-linked immunosorbent assay (Euroimmun, Lübeck, Germany) according to the manufacturer’s instructions. This test provides a numerical value (ratio) reflecting the luminescence intensity, which is a surrogate for the amount of IgG antibodies. We followed Euroimmun’s recommendation of interpreting a ratio equal to or greater than 1.1 as a positive test result. Antibody levels were subsequently converted to Binding Antibody Units (BAU/ml) according to the World Health Organization International Standard [7].

In a subgroup of patients, specific T-cell responses were evaluated using the TIGRA-test® (Generium, Russia). For this test, peripheral blood mononuclear cells were isolated from whole-blood samples by centrifugation with a 1.077 Ficoll gradient and incubated with SARS-CoV-2 structural peptides spike (S) and nucleocapside (N) overlapping the assay plates. If specific CD4^+^ and CD8^+^ cytotoxic lymphocytes were present in the blood, they emitted interferon-γ after contact with the antigen. The results of the test were T-spot responses to SARS-CoV-2 structural peptides S and N, which were estimated separately. According to the manufacturer’s instructions, counts >12 spots per 340,000 blood mononuclear cells were considered positive test results. The test results were interpreted as indeterminate if >14 spots were counted in a plate with pure AIM medium (negative control).

### Ethics

The study was conducted in accordance with the principles of the Declaration of Helsinki. All participants provided written informed consent to participate in the study. All study procedures were approved by the Biomedical Ethics Board of the Saint Petersburg State University Hospital (protocol no. 11/20 from November 19, 2020). The study protocol was registered at www.clinicaltrials.gov (NCT 04633915).

### Statistical analysis

Normally distributed quantitative data are presented as means ± standard deviations, whereas parameters with non-Gaussian distribution are expressed as medians and interquartile ranges (*Q*_1_–*Q*_3_). Absolute values and percentages are used to describe categorical data. Correlations were analyzed using Spearman’s rank correlation coefficient (GraphPad Prism v.9.0.0).

Since the semi-quantitative tests provided numerical values, we analyzed these data quantitatively. As the observations were clustered and the matrix had single missing values, we assessed the dynamics of IgG levels in patients at different time points using a linear mixed-effects model (analysis of variance), wherein the fixed effects were "time,” “group,” and the “time × group” interaction and the random effect was "id" (patient): lmer(IgG_bc ∼ time + group + time × group+(1|id). The analysis was performed using R v.4.1.1, and the "lme4" package. We calculated the statistical significance of the fixed effects using the Satterthwaite approximation (lmerTest package) because the calculation of *P* values was not implemented in the lme4 software package. *P* values <0.05 were considered statistically significant. Pairwise comparisons were performed using Tukey’s post hoc test. Since the assumption of homoscedasticity was not met, the Box–Cox transformation was performed (the "boxcox" function in the package "MASS"). The transformed values were used for the analysis.

Reasonably, age modified the strength of the humoral response. Indeed, the association between age and antibody levels was different between the two groups at all time points (Supplementary Figure 1). We built a second model that included the «age × group» interaction, lmer(IgG_bc ∼ time + group + time × group + age × group+(1|id).

## Results

The patient demographics are summarized in Table 1. Three participants received immunosuppressive drugs: one HD patient took 10 mg of prednisone a day due to systemic vasculitis, one HD patient and one subject among controls took topical steroids for asthma maintenance treatment. Patients receiving HD were older and had more comorbidities compared with the control group. The incidence of COVID-19-related symptoms did not differ between the groups, except for the loss of smell, which occurred much more frequently among the controls. The percentage of asymptomatic cases was comparable between the groups.

**Table 1.** Demographic and clinical characteristics of patients receiving hemodialysis and controls at baseline.

Factors	HD patients (*n* = 23)	Non-HD (controls) (*n* = 27)	*p* value
Age, years	55 ± 16	39 ± 8	<0.0001
Sex (male/female)	17/6	14/13	0.19
BMI, kg/m^2^	24.5 ± 4.8	26.8 ± 4.2	0.08
Comorbidity, CIRS scores	14 [11;16]	1 [0;3]	<0.0001
Diabetes	5 (21.7%)	2 (7.4%)	0.23
Autoimmune disease	2 (8.7%)	0	0.27
Immunosuppressive drugs	2 (8.7%)	1 (3.7%)	0.59
*Cause of ESKD (only for HD patients)*		–	–
Glomerulonephritis (primary or secondary)	5 (21.4%)	–	–
Hypertensive kidney disease	4 (17.4%)	–	–
Diabetic nephropathy	4 (17.4%)	–	–
Hereditary kidney disease	4 (17.4%)	–	–
Other/miscellaneous	6 (26.1%)	–	–
Dialysis vintage, months	41 [29; 71]	–	–
Duration of COVID-19, day	17.2 ± 4.8 (from 9 to 25)	17 ± 5.9 (from 5 to 30)	0.88
*COVID-related symptoms*			
cough	10 (43.5%)	13 (48%)	0.78
Shortness of breath	7 (30.4%)	7 (26%)	0.76
Temperature	20 (87%)	23 (85%)	0.99
Sore throat	5 (21.7%)	5 (19%)	0.99
Anosmia	7 (30.4%)	19 (70%)	0.01
Completely asymptomatic	3 (13%)	1 (4%)	0.32

Normally distributed data are expressed as means ± standard deviations, data with a skewed distribution are presented as medians, first and third quartiles. Categorical values are presented as absolute numbers (percentages). BMI: body-mass index; CIRS: cumulative illness rating scale; ESKD: end-stage kidney disease; HD: hemodialysis.

Descriptive statistics of immunogenicity for the groups are presented in the Table 2. All the participants had positive anti-SARS-CoV-2 IgG levels at baseline. The seropositivity rate declined in non-renal controls over time and was 85% (23 of 27) and 70.4% (19 of 27) at weeks 18 and 26, respectively. In contrast, all patients receiving HD remained seropositive by the end of the study. Thus, the risk of seropositivity at week 26 was consistently greater among patients receiving HD than that in the control group: relative risk (RR)=1.4 [95% confidence interval (CI): 1.17–1.94] (reciprocal of RR = 0.7 [95% CI: 0.52–0.86]), *p* = 0.0064.

**Table 2.** SARS-CoV-2 IgG levels and T-spot counts at different time points in patients receiving hemodialysis and non-renal controls.

Characteristic	Mean (SD)	95%CI	Median [*Q*1–*Q*3]
IgG, BAU/ml			
HD patients. 10 weeks	154.2 (55.6)	130.2; 178.2	165.8 [104; 195.4]
HD patients. 18 weeks	129.7 (50.3)	107.4; 152	121.9 [95; 158.5]
HD patients. 26 weeks	135.2 (67.2)	103.8; 166.6	125.8 [87; 177.5]
Controls. 10 weeks	121.4 (66.4)	95.1; 147.7	99.2 [71.2; 140.8]
Controls. 18 weeks	88.7 (49.8)	69; 108.4	80.6 [42.9; 116.6]
Controls. 26 weeks	68.5 (44.3)	51; 86	63.4 [32.6; 85.9]
Spots count			
HD patients. 10 weeks	88.5 (49.4)	86.5 [56.2; 119]	60; 117
HD patients. 18 weeks	79.5 (52.4)	65 [51; 95]	47.8; 111.2
HD patients. 26 weeks	108 (59.4)	92 [56.5; 162]	68.1; 147.9
Controls. 10 weeks	52.8 (31)	43 [27.5; 69.5]	38.3; 67.3
Controls. 18 weeks	55.8 (28.5)	51.5 [42; 65.5]	42.5; 69.1
Controls. 26 weeks	54.1 (24)	52.5 [36.5; 65]	42.9; 65.3

HD: hemodialysis; IgG: immunoglobulin G; BAU: binding antibody units.

**Figure 1.**
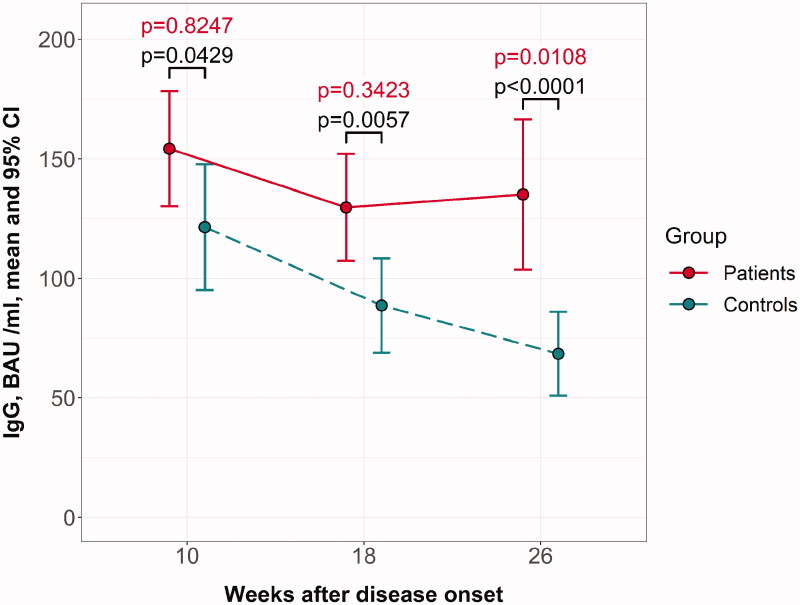
Dynamics of SARS-CoV-2 IgG S1/S2 antibodies until 26 weeks after diagnosis of COVID-19 in patients receiving hemodialysis and non-renal controls. *P* values for post hoc pairwise comparisons in a model that includes the time, group, time × group interaction, and subject (random effect) are depicted in black; red color shows *P* values for post hoc pairwise comparisons in an age-adjusted model, including the time, group, time × group interaction, age × group interaction, and subject (random effect).

Specific SARS-CoV-2 IgG levels differed between the groups at week 10 with significance close to the borderline (*p* = 0.0429) but were significantly lower in the controls than in adults receiving HD at week 18 (*p* = 0.0057) and week 26 (*p* < 0.0001) (Figure 1). In both groups, IgG levels decreased from week 10 to week 26, however antibodies vanished more rapidly in the controls than in the dialysis-dependent group (analysis of variance *p* = 0.0012 for the “time × group” interaction) (Figure 1). Age may have had a significant impact on the humoral response, and we observed a different relationship between age and antibody levels in the two groups (Supplementary Figure 1). Therefore, we performed pairwise post-hoc comparisons for the age-adjusted model. Twenty-six weeks post-diagnosis, the differences between the groups were statistically significant (*p* = 0.0108).

**Figure 2.**
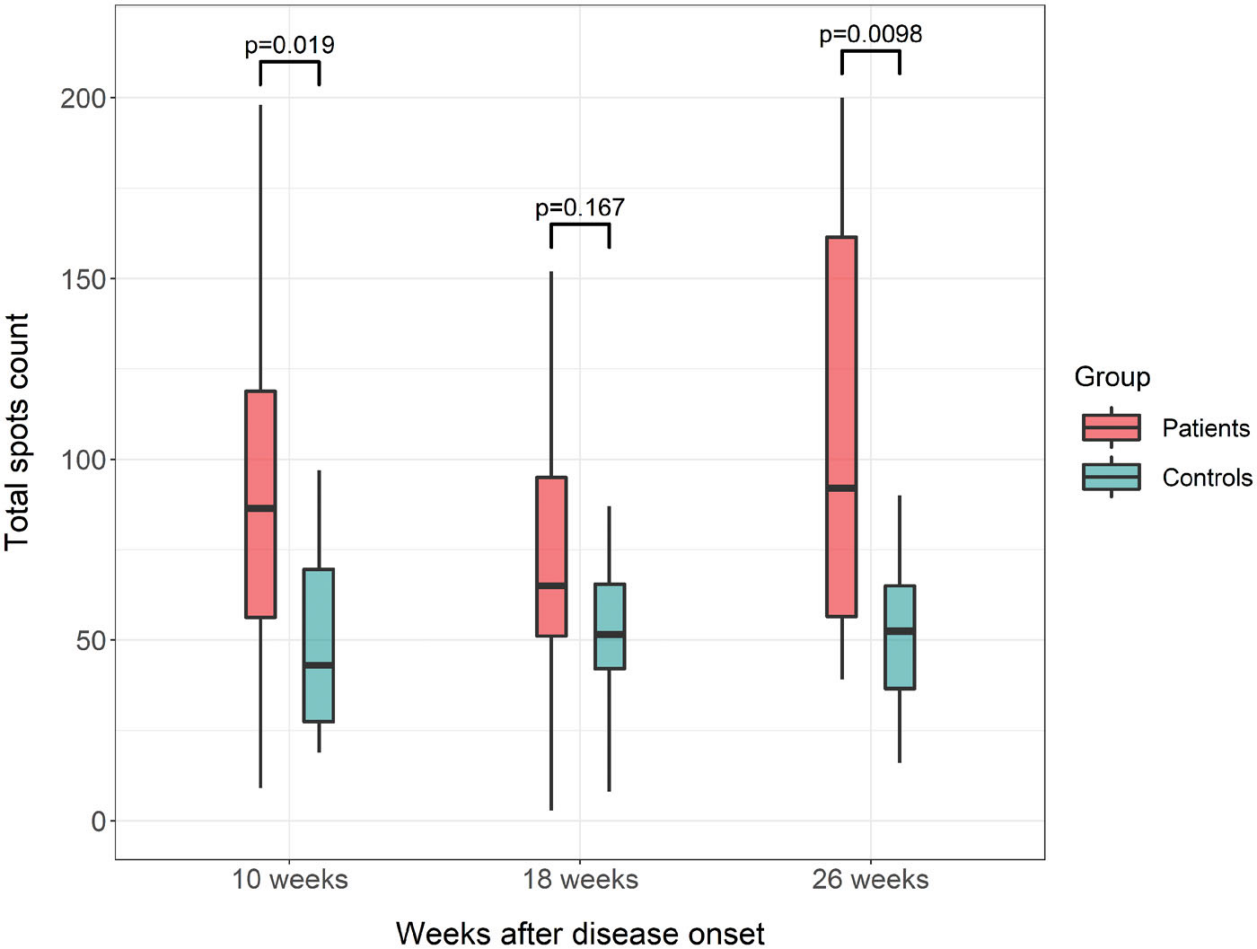
Evolution of specific CD4^+^ and CD8^+^ T-lymphocytes until 26 weeks after the diagnosis of COVID-19 in patients receiving hemodialysis and non-renal controls.

In subgroup analysis, the cellular response was evaluated in 14 patients receiving HD and 20 controls. Initially, the *t* test result was positive in all non-renal subjects, while one patient receiving HD had a negative test result. At the end of the study, all the participants showed positivity in terms of specific T-cell responses, except for one patient receiving HD with indeterminate test results. The total count of T-spots (a sum of spots to both spike and nucleocapside structural peptides) was significantly lower in the control group than in patients receiving HD at weeks 10 (*p* = 0.019) and 26 (*p* = 0.0098) after COVID-19 diagnosis, whereas this finding did not reach statistical significance at week 18 (Figure 2).

We observed no statistically significant correlations between humoral and cellular response at week 10 (*p* = 0.17) and week 18 (*p* = 0.1). There was a statistically significant correlation between IgG levels and T-spots at week 26 (ρ = 0.38 [95%CI: 0.02-0.66], *p* = 0.033) and over the entire observational period (ρ = 0.27 [95%CI: 0.07–0.45], *p* = 0.0074), although it was weak in both cases. There were no correlations between the time for viral clearance and the magnitude of neither humoral nor T-cell response in both groups.

## Discussion

The durability of the humoral immune response after natural COVID-19 in patients receiving maintenance HD has been investigated previously in several studies [3–5], which were mostly retrospective in their nature. Overall, antibody response was shown to be sustained over time, with a long-term seropositivity rate of 75% to 94% [4,8]. In this study, we investigated the 6-month kinetics of SARS-CoV-2 IgG antibody and specific CD4^+^ and CD8^+^ T-lymphocyte levels in patients receiving HD compared with those in individuals without preexisting renal diseases. Hence, our study is, to best of our knowledge, the first that answers the question whether the long-term immune response in patients with ESKD is comparable to individuals with normal kidney function.

Patients with chronic kidney disease (CKD) are commonly known to have altered humoral and cellular immune responses [9]. For instance, seropositivity rate after hepatitis B immunization has been shown to decline along with CKD progression. Furthermore, patients with CKD have demonstrated a decreased antibody response to the pneumococcal vaccine compared with healthy controls [10]. In accordance with existing knowledge, we supposed that SARS-CoV-2 IgG antibodies wane more rapidly in patients receiving HD than in controls. However, this hypothesis was not confirmed: in contrast, SARS-CoV-2 antibody levels declined more rapidly in non-renal participants. Forbes et al. examined the antibody response following COVID-19 in 122 patients receiving HD and found that patients with underlying diabetes and current immunosuppression demonstrated the positive slope over time [11]. However, prevalence of these two conditions did not differ between groups in our study. Certain studies discovered increased age as an independent predictor of greater IgG antibody response after COVID-19 in the general population [12,13], and this could at least partially explain our results. Nevertheless, in our cohort the magnitude of humoral immunity remained higher in patients receiving HD even after adjustment for age.

In the recent study Cappuccilli at al. found no differences between seropositivity rates after around 6 months from COVID-19 in the mixed cohort of HD and renal transplant recipients, and in the subjects with normal renal function. However, the study was primarily focused on the comparison of SARS-CoV-2 humoral immunity between dialysis and renal transplant recipients, and authors did not compare neither the magnitude nor the dynamics of humoral responses in renal and non-renal groups [14].

Another finding of our study was the durable cellular response in patients receiving HD, which was even stronger than that in the control group. The ability of patients receiving dialysis to generate COVID-19-specific T-cells shortly after disease comparable or even higher than in healthy was previously investigated by Anft et al. [15]. It is important to note that efficiency of cellular immune response was confirmed by CD4^+^ and CD8^+^ T-cells multiply cytokine production.

This study had some limitations. First, the sample size was small. Second, since the protective antibody levels and T-cell counts are still unknown, the practical implications of our findings may be limited. Third, one patient receiving HD had an indeterminate *t* test result by the end of the study. This result may indicate an increased level of spontaneous interferon-γ production by T-lymphocytes. This can occur in the presence of either an acute phase of the infection process or a chronic inflammatory or autoimmune process, in which an adequate immune response is not formed [16]. Nevertheless, considering the results of the study by Borekci et al. [17], we did not exclude these data from the analyses. Fourth, as the time of follow-up was limited to 6 months, the exact time of immunity loss remains unclear. Fifth, we didn’t perform neutralization antibody tests, which are better predictive of humoral immune protection.

In summary, compared with non-renal subjects, patients receiving HD maintain significant long-term humoral and cellular immune responses following natural COVID-19. Our data are encouraging that HD patients should develop and maintain similar response to vaccination compared to controls. These findings may have important implications in vaccination boosting strategies for patients receiving maintenance HD. To date, a protective antibody titer still remains to be established, and vaccination is strongly needed regardless of a history of previous COVID-19, especially in the settings of the rapid spread of novel SARS-CoV-2 variants. According to available data, vaccination elicits a more robust humoral and cell-mediated immune response than that of natural infection [18]. We found no evidence that HD patients who recovered from natural COVID-19 require adjustment of vaccination programs, i.e., high doses or more vaccine shots (as it is recommended for hepatitis B immunization). Since there is no sufficient data on the optimal timing between prior COVID-19 and vaccination, similar recommendations should be followed for HD patients as for the general population.

## Supplementary Material

Supplemental Material

## Data Availability

The data of the study, including the code used in the analyses, are available from the corresponding author upon reasonable request.
